# Cost-effectiveness of option B+ in prevention of mother-to-child transmission of HIV in Yunnan Province, China

**DOI:** 10.1186/s12879-019-3976-5

**Published:** 2019-06-11

**Authors:** Xiaowen Wang, Guangping Guo, Jiarui Zheng, Lin Lu

**Affiliations:** 1 Yunnan Center for Disease Control and Prevention, No 158, Dongsi Street, Xishan District, Kunming, Yunnan Province China; 2 Kunming Medical University, No. 1168, west Chunrong Street, Chenggong district, Kunming, Yunnan Province China; 3 Yunnan Maternal and Child Health Care Hospital, No. 200, Gulou Street, Wuhua District, Kunming, Yunnan Province China; 4 Health Commission of Yunnan Province, No. 309, Guomao Street, Kunming, Yunnan Province China

**Keywords:** Option B +, HIV prevention, Cost-effectiveness analyses, Decision making

## Abstract

**Background:**

Although Option B+ may be more costly than Options B, it may provide additional health benefits that are currently unclear in Yunnan province. We created deterministic models to estimate the cost-effectiveness of Option B+.

**Methods:**

Data were used in two deterministic models simulating a cohort of 2000 HIV+ pregnant women. A decision tree model simulated the number of averted infants infections and QALY acquired for infants in the PMTCT period for Options B and B+. The minimum cost was calculated. A Markov decision model simulated the number of maternal life year gained and serodiscordant partner infections averted in the ten years after PMTCT for Option B or B+. ICER per life year gained was calculated. Deterministic sensitivity analyses were conducted.

**Results:**

If fully implemented, Option B and Option B+ averted 1016.85 infections and acquired 588,01.02 QALYs.The cost of Option B was US$1,229,338.47, the cost of Option B+ was 1,176,128.63. However, when Options B and B+ were compared over ten years, Option B+ not only improved mothers’ten-year survival from 69.7 to 89.2%, saving more than 3890 life-years, but also averted 3068 HIV infections between serodiscordant partners. Option B+ yielded a favourable ICER of $32.99per QALY acquired in infants and $5149per life year gained in mothers. A 1% MTCT rate, a 90% coverage rate and a 20-year horizon could decrease the ICER per QALY acquired in children and LY gained in mothers.

**Conclusions:**

Option B+ is a cost-effective treatment for comprehensive HIV prevention for infants and serodiscordant partners and life-long treatment for mothers in Yunnan province, China. Option B+ could be implemented in Yunnan province, especially as the goals of elimination mother-to-child transmission of HIV and “90–90-90” achieved, Option B+ would be more attractive.

**Electronic supplementary material:**

The online version of this article (10.1186/s12879-019-3976-5) contains supplementary material, which is available to authorized users.

## Background

The recommendations of the World Health Organization (WHO) on the prevention of mother-to-child transmission (PMTCT) of HIV infection have evolved significantly over time. Up to 2011, the WHO recommended that all pregnant women with HIV infection and CD4 count below 350 cells/mm^3^ initiate antiretroviral therapy(ART) at gestation week 14 and continue until one week post-partum. This strategy became known as PMTCT Option A [1, 2]. In the same year, the guidelines also commanded the other strategy, Option B, which extended ART for mothers out through the end of breastfeeding [1, 2]. Then, in light of new, compelling evidence that early ART improved treatment outcomes and sustained viral suppression reduced odds of onward sexual transmission [3–7], the WHO introduced a new strategy in 2013, Option B+,which utilized a new ART. PMTCT Option B+ is providing life-long ART to all pregnant women who once tested HIV positive regardless of CD4 counts or clinical stage [8]. In theory, Option B+ was expected to not only achieve the Global Plan Target of new pediatric HIV infections elimination in 2015, but also achieve the universal access to HIV treatment for mothers to keep mother alive [2].

Option B+ has already been adopted by a range of low- and middle-income countries, including Malawi, Zambia,Tanzania, South Africa and Kenya [1, 6, 8, 9]. Early studies indicate the incremental cost of switching from Option B to Option B+ in PMTCT programs ranged from US$92,813 to US$605,739 per 1000 women [10] and the Incremental cost-effectiveness ratio (ICER) was US$1370 per year of life saved compared with Option B [3] and ranged from US$6000 to US$23,000 per infection averted compared with Option A [6]. Although the most analyses have identified the likely cost-effective of Option B+, a recent review of published cost-effectiveness analyses of Option B+ for prevention of mother-to-child transmission of HIV in developing countries demonstrated whether Option B+ was dominant, cost-effective or non cost-effective depended on the differences of the decision model structure and input parameter values. So decision makers still need additional analyses of model to inform the local funding decision [11].

PMTCT programs became a key public health priority in China in 2002 [12] and Option B+ began to implement in PMTCT programs in 2015 [13]. China’s Yunnan Province, currently has the highest prevalence of HIV infection nationwide. PMTCT programs have been operating since 2003. It’s reported that HIV prevalence in antenatal care (ANC) in Yunnan province is estimated at 0.3%, which translates to approximately 2000 new HIV-exposed infants born to pregnant women with HIV each year. Because HIV counseling and testing has been included in ANC nationwide in China since 2011 [14], and because there is nearly 100% uptake of ANC in Yunnan, pregnant women who have HIV infections are relatively easily identified. Next step, the effective ART is critical. Presently, Option B + is also offered to HIV-infected pregnant women in Yunnan, which means the more expensive second-line ART and life-long ART. Option B+ may be more costly than Option A and Option B while it may provide more health benefits, but it keeps unclear currently in Yunnan province. Therefore, we aimed to project the clinical outcomes and cost-effectiveness of the Option B+ from short-term and long-term respectively in Yunnan Province so as to help inform the funding evidence for the decision making of the optimal ART regimen for PMTCT and HIV/AIDS prevention and therapy.

## Methods

The methodology we applied in the study was in accordance with the Consolidated Health Economic Evaluation Reporting Standards (CHEERS) statement guidelines [15].

Based on the data from the information system of preventing mother-to-child transmission of HIV, Syphilis and Hepatitis B in Yunnan maternal and infant health care hospital, a hypothesis cohort of HIV-infected women was estimated with the number of 2000 (720,435 pregnant women were tested and HIV prevalence rate in pregnant women was about 0.3%). The mean age of pregnant women with HIV at first ANC visit was 25 years. Mean CD4 count was 445 (36% of women with CD4 count≤350 cell**/**μl). Among them 1120 chose to delivery (the termination rate was about 44%).

### The cost-effectiveness of option B+ in short-term

We defined the short-term of the study as the period of prevention mother-to-child transmission of HIV (from the first ANC to the period of infants with 18 months old).We examined two strategies in our analyses: (1) WHO Option B(comparator), (2) WHO Option B+. These two strategies are compared in Table 1.

**Table 1:** Regimens of Option A, Option B and Option B+ recommended by WHO

	Option B	Option B+
Mother	women received ART during pregnancy if eligible by either CD4 or clinical criteria. Women not eligible for ART were modelled to receive triple-antiretroviral prophylaxis of zidovudine, lamivudine and lopinavir and ritonavir (AZT* + 3TC* + LPV/r*) or zidovudine, lamivudine and efavirenz (AZT + 3TC + EFV*) from 14 weeks until 42 days after delivery	all women received lifelong ART
Infant	Daily NVP* or twice daily AZT from birth until 42 days of age	Daily NVP or twice daily AZT from birth until 42 days of age

*NVP Nerirapine, *AZT* Zdovudine, *3TC* Lamivudine, *LPV/r* Lopinavir and Ritonavir, *EFV* Efavirenz

All the analyses was from the health care system perspective. Clinical outcomes included number of pediatric infections averted and quality adjusted life-years (QALY) acquired for infants. Economic outcomes included ANC costs, cost of ART, delivery cost and cost of infant formula feeding, cost of infant prophylaxis and early infant diagnosis. Incremental cost-effectiveness ratio (ICER) per pediatric infection averted and per QALY acquired, in US dollars (US$) in 2016, were calculated. We calculated QALY by weighting with health utilities of 0.74. All costs and health outcomes were discounted at 3% per year. We used WHO guidance as criteria to interpret cost-effectiveness [3]. The net number of QALY acquired by a single averted paediatric HIV infection was a function of the difference between the expected number of QALY of a child without HIV infection and the excepted number of QALY of a child with HIV infection. We adopted the health utilities of 0.74 for the HIV sample [16] and of 1 for the general sample to weight the life years gained and the determined QALY acquired. An intervention was considered cost-effective compared with the next least-expensive alternative if its ICER was less than 3 times the 2016 Yunnan Province per capita gross domestic product (GDP, US$ 4222) [17], or US$ 13,000 per life-year or QALY acquired.

Based on the applicability of simple decision tree, which are most useful to model events or health status without occurring repeatedly and the likelihood of events occurring in the model keeps unchanged over time [18]. Decision tree model was used to do the analyses. We modeled ANC coverage at100%, HIV testing and counselling coverage within ANC at 100% and adherence to PMTCT regimens for women and infants all at 100% according to the reality of Yunnan province [19]. To demonstrate the features of every strategy, all women in the base-case analyses were assumed to be identified as HIV-infected at their first ANC visit.

Among parameters related to the analyses, the rate of mother-to-child transmission of HIV for Option B and Option B+ were from the surveillance data of Yunnan province, 3.86 and 3.86% respectively. All the cost in the PMTCT period recommended by the surveillance data of Yunnan Maternal and Infant Health Care Hospital. The cost of rapid HIV testing was US$4.06 per test and all pregnant women were assumed to undergo at least one HIV test during the first antenatal visit for Option B and Option B+. Option B to determine the eligibility for ART at a cost of US$ 13.06 per test and 4 times in the PMTCT period. Costs of ARVs for Option B included 6 months during pregnancy and 42 days after delivery. The cost of drugs per woman receiving B and B+ in the PMTCT programs was US$253.79 per woman (consisting of US$235.65 for maternal ART, US$18.14 for infant ARVs prophylaxis). The cost of ART per year was derived from a cost of ART study including Yunnan province in China [19].

In the sensitivity analysis, efficacy of Option B/Option B+ in reducing transmission rates were varied using ranges reported in the literature. We also took into account the lifetime ART cost for infants with HIV.

**Fig. 1:**
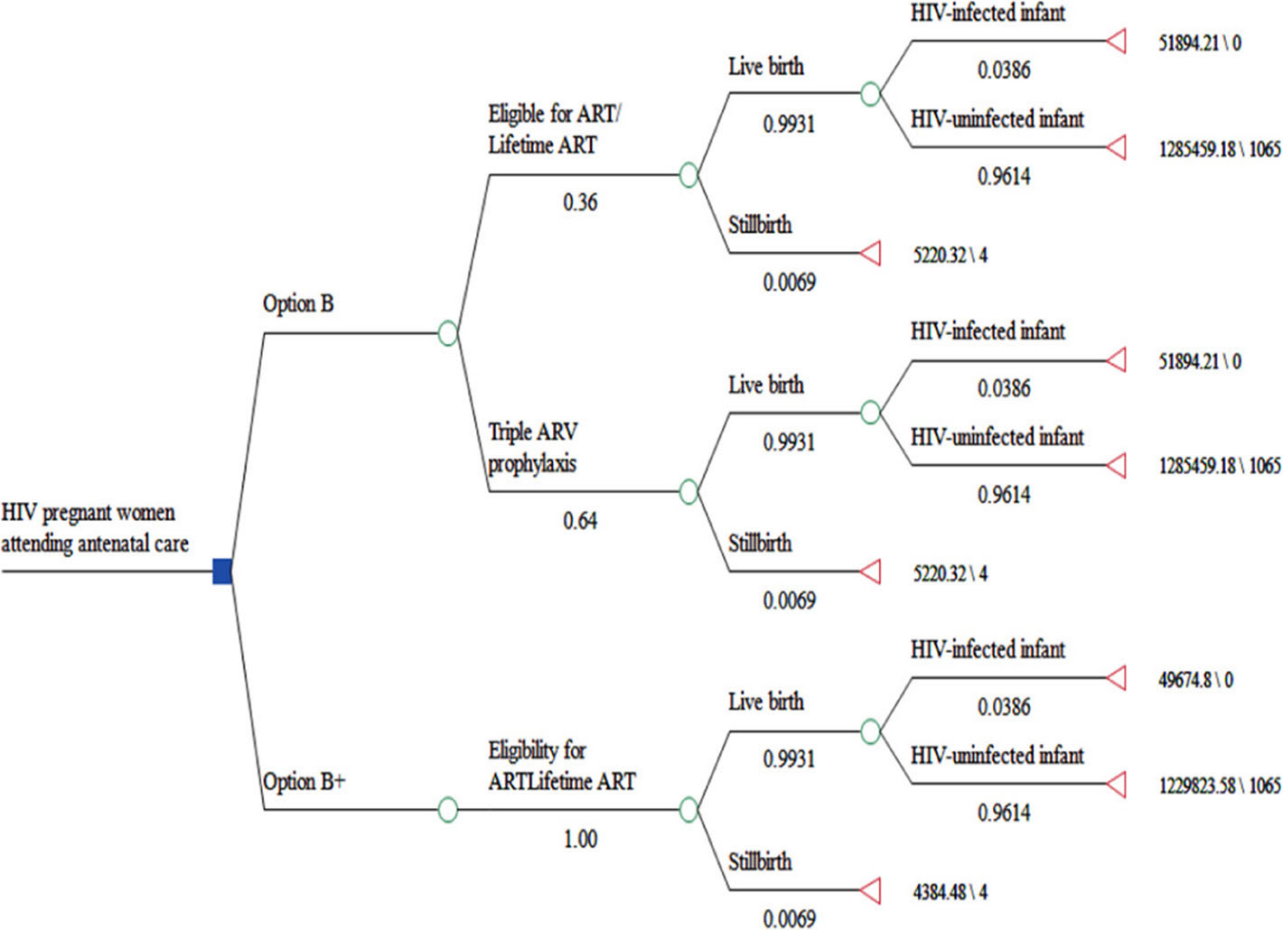
Simple decision tree model for infant health outcomes

All the parameters related to disease progression, ART regimens, and cost in base case analysis and sensitivity analysis were showed in Table 2 and Additional file 1. The simple decision tree used for modeling infant outcomes is shown in Fig. 1.

**Table 2:** Input parameters and plausible ranges used for sensitivity analysis in the simple decision tree analysis for infant outcomes

Parameters	Base-case	Reference
HIV Epidemiology		
Number of HIV-infected pregnant women	2000	Surveillance data
Percentage of pregnant women with CD4 count> 350 cells/μl(%)	64	Surveillance data
MTCT transmission rates		
Background transmission rate without intervention (%)	34.80	Surveillance data
Transmission rate, Option A with infant prophylaxis, formula feeding(%)	7.28	Surveillance data
Transmission rate, Option B, Option B+ and eligible women on ART With infant prophylaxis, formula feeding(%)	3.86(1.00–5.00)	Surveillance data, [20]
Delivery proportion(%)	55.79	Surveillance data
Newborn mortality rate	0.0069	China Health Statistical Yearbook 2013
Health utility	0.74	[21]
Cost		
HIV testing and counselling	US$4.06	Surveillance data
CD4 screening	US$52.24	Surveillance data
Drugs in Option A	US$23.94	Surveillance data
Drugs in Option B and Option B+(until 42 days after delivery)	US$235.65	Surveillance data
Infant ARVs prophylaxis	US$18.14	Surveillance data
Formula feeding	US$580.42	Surveillance data
Infant diagnosis	US$26.12	Surveillance data
Discounted lifetime cost for an HIV infected child on ART(69.5 years)	US$247,163.94	[19]

### The cost-effectiveness of option B+ in long-term

**Fig. 2:**
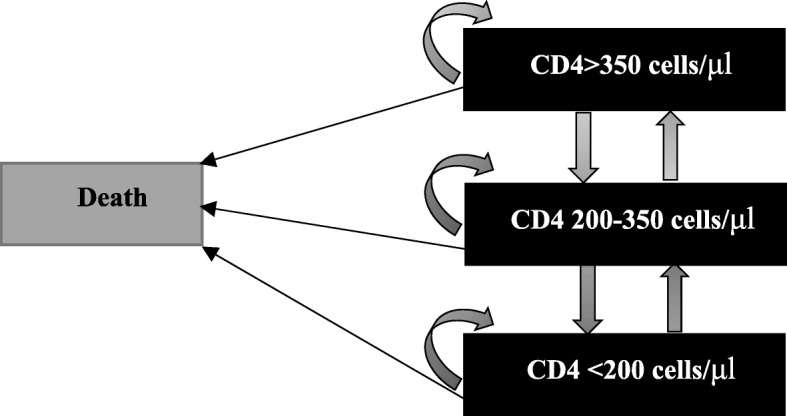
Markov status used in the long-term analyses

We defined the long-term as the 10 years starting from the date of PMTCT services concluded for the 2000 pregnant women. Based on the applicability of Markov modeling, which was mostly used for more complex events occurring over time [22]. A Markov decision model was used to simulate a cohort of 2000 pregnant women living with HIV. After entering the model and receiving one of the ART regimens (Option B—ART eligibility based on CD4 count, or Option B + —lifelong ART regardless of CD4 count), subsequent “movement” through three health states was defined by CD4 cell count levels and a death state (as the absorbing state, see Fig. 2). We estimated one month as a “cycle”, a total 120 “cycles” were calculated in 10 years. Under the different ART regimens, the pregnant women living with HIV have different CD4+ transforming stages, meanwhile, the women living with HIV at different CD4+ levels have different transmission probability to their serodiscordant partners. The Markov decision model simulated the mother life-years saved andQALY acquired (life years were weighted by the health utility classified by CD4+ count level reported by a published study [23]), serodiscordant infections averted(calculated by the sex acts per month with regular partner reported by a publish study in Yunnan province [21]), and costs of treatment for mothers for 10 years after finishing PMTCT services. Incremental cost-effectiveness ratio (ICER) per life year gained and per QALY acquired were calculated in US dollars (US$) in 2016. All costs and health outcomes were discounted at 3% per year. We also used WHO guidance as criteria to interpret cost-effectiveness [3]. The detail progression of the modeling listed in Additional file 1.

**Fig. 3:**
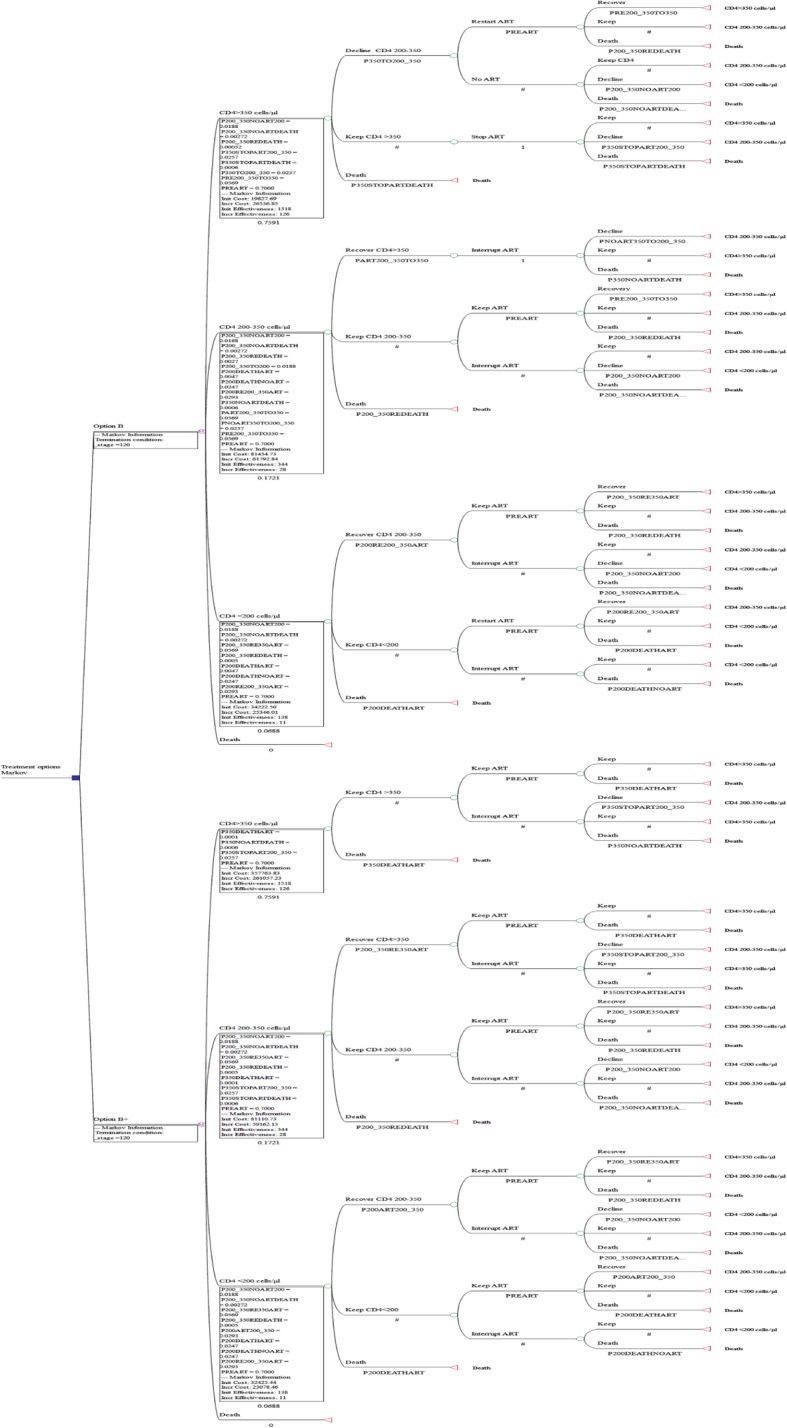
Markov decision tree for the health outcomes of pregnant women living with HIV

In sensitivity analysis, time horizons (5 years, 20 years), and ART coverage rates (45, 90%) were specially investigated. All input parameters used in the Markov decision tree model and plausible ranges used for the sensitivity analysis are described in Table 3. The final Markov decision model is shown in Fig. 3 and all variable assignments are described in Table 4.

**Table 3:** input parameters and plausible ranges used for sensitivity analysis in the Markov decision tree analysis for maternal and serodiscordant partner outcomes

Parameters	Base-case	Reference
Primary probability		
CD4 counts at the last testing of PMTCT period		Surveillance data
Percentage of pregnant women with CD4 count≧350 cells/μl(%)	75.91	
Percentage of pregnant women with CD4 count 200–350 cells/μl(%)	17.21	
Percentage of pregnant women with CD4 count< 200 cells/μl(%)	6.88	
Death probability		
Monthly probability death of off ART, CD4 200-350cells/μl (α_11_)	0.00272	[16, 23, 24]
Relative probability death per month off ART, CD4 > 350 vs. 200-350cells/μl (α_21_:α_11_)	0.206(0.206–0.258)	
Relative probability death per month off ART, CD4 < 200 vs. 200-350cells/μl (α_31_:α_11_)	9.08(3.45–9.08)	
Relative probability death on vs. off ART for the same CD4 counts (α_i2_:α_i1_)	0.19(0.14–0.25)	
Transmission probability		[23]
Off ART, monthly probability of moving from CD4 > 350 to 200–350 cells/μl	0.0257(0.0119–0.0289)	
Off ART, monthly probability of moving from CD4 200–350 to < 200 cells/μl	0.0188(0.0186–0.0274)	
On ART, monthly probability of moving from CD4 200–350 to > 350 cells/μl	0.0569(0.0247–0.0888)	
On ART, monthly probability of moving from CD4 < 200 to 200–350 cells/μl	0.0293(0.0274–0.0683)	
Per vaginal sex act probability of HIV transmission from women to man		[25]
CD4 > 350cells/μl	0.02(0.01–0.04)	
CD4 200-350cells/μl	0.03(0.01–0.04)	
CD4 < 200cells/μl	0.05(0.03–0.06)	
% efficacy of ART in reducing HIV transmission	92(26–99)	[23]
QALY weight HIV-positive on ART or CD4 > 350cells/μl	0.947	[23]
QALY weight HIV-positive CD4 200–350 cells/μl	0.799	[23]
QALY weight HIV-positive CD4 < 200 cells/μl	0.453	[23]
Sex acts per month with regular partner	1–2	[21]
Cost parameters		
Provider unit cost ART per year (2016,US$)	3561	[19]

**Table 4:** variable assignment in Markov decision tree

Variable name	Meaning standed for	Base value	Lower value	Upper value
P350TO200_350	NO ART, monthly probability of moving from CD4 > 350 to 200–350 cells/μl	0.02570	0.02890	0.01190
PREART	Coverage rate of ART	0.70000	0.45000	0.90000
PRE200_350TO350	On ART, monthly probability of moving from CD4 200–350 to > 350 cells/μl	0.05690	0.02470	0.08880
P200_350REDEATH	Monthly probability death of on ART, CD4 200-350cells/μl	0.00052	0.00010	0.00068
P200_350NOART200	STOP ART, monthly probability of moving from CD4 200–350 to < 200 cells/μl	0.01880	0.01860	0.02740
P350STOPART200_350	STOP ART, monthly probability of moving from CD4 > 350 to 200–350 cells/μl	0.02570	0.01190	0.02890
P350STOPARTDEATH	Monthly probability death of stopping ART, CD4 > 350cells/μl	0.00060	0.00060	0.00070
P200_350TO200	No ART, monthly probability of moving from CD4 200–350 to < 200 cells/μl	0.01880	0.01860	0.02740
P200RE200_350ART	On ART, monthly probability of moving from CD4 < 200 to 200–350 cells/μl	0.02930	0.02740	0.06830
P200DEATHART	Monthly probability death of on ART, CD4 200 cells/μl	0.00470	0.00130	0.00620
P200DEATHNOART	Monthly probability death of off ART, CD4 200 cells/μl	0.02470	0.00930	0.02470
P200_350NOARTDEATH	Monthly probability death of off ART, CD4 200-350cells/μl	0.00272	0.00000	0.00272
PART200_350TO350	On ART, monthly probability of moving from CD4 200–350 to > 350 cells/μl	0.05690	0.02470	0.08880
PNOART350TO200_350	No ART, monthly probability of moving from CD4 > 350 to 200–350 cells/μl	0.02570	0.01190	0.02890
P350NOARTDEATH	Monthly probability death of off ART, CD4 > 350cells/μl	0.00060	0.00060	0.00070
P200ART200_350	Starting ART, monthly probability of moving from CD4 < 200 to 200–350 cells/μl	0.02930	0.02740	0.06830

We used TreeAge Pro2016 to perform all statistical analyses.

## Results

### Cost-effectiveness analyses: infant health outcomes in the short-term

Table 5 shows the cost, outcomes, and cost-effectiveness of the two different strategies modelled via the decision tree model to prevent new infant infections. If fully implemented, Option B and Option B+ averted 1016.85 infections and acquired 588,01.02 QALYs.The cost of Option B was US$1,229,338.47, the cost of Option B+ was 1,176,128.63. Option B+ made a minimize cost when compared with Option B.

**Table 5:** Cost-effectiveness analyses of infant outcomes in the short-term

	Option B	Option B+
Cost		
Program Cost (Total 18 months)	US$1,229,338.47	US$1,176,128.63
Pediatric outcomes		
Expected number of infection averted	1016.85	1016.85
QALY averted	58,801.02	58,801.02
Cost-effectiveness ratios		
Cost per infection averted	US$1208.96	US$1156.63
Cost per QALY acquired	US$20.91	US$20.00
Cost minimization in infection averted (compared to Option B)	–	Option B+
Cost minimization in QALY acquired(compared to Option B)	–	Option B+

### Sensitivity analysis for infant health outcomes

Table 6 shows the results of our sensitivity analyses. For infant outcomes, Option B+ dominated as the most-effective strategy for the prevention of new pediatric infections and for the QALY acquisition for infants when compared with Option B.

**Table 6:** Sensitivity analysis for infant outcomes

Model parameters	OptionB	OptionB+
US$/Infection averted		
Cost of ART(US$)		
including the lifetime ART cost of infant(69.5 years)	1608.37	1557.29
efficacy of Option B/Option B+ in reducing transmission rates		
Best case-1.00%	1207.50	1155.23
Worst case-5.00%	1209.57	1157.22
US$/QALY aquired		
Cost of ART(US$)		
including the lifetime ART cost of infant(69.5 years)	27.81	26.93
efficacy of Option B/Option B+ in reducing transmission rates		
Best case-1.00%	20.89	19.98
Worst case-5.00%	20.92	20.01

### Cost-effectiveness analyses: maternal and serodiscordant partner outcomes in the long-term

Compared to Option B, Option B+ had an ICER per life-year gained and QALY acquired for mothers was US$ 5183.96 and US$ 5355.42 respectively. However, when we included the benefit of serodiscordant partners over 10 years, 410 infections were averted with Option B at a cost per infection averted of US$ 8098.99, and 3068 infections were averted with Option B+ at a cost per infection averted of US$ 7655.70.

**Fig. 4:**
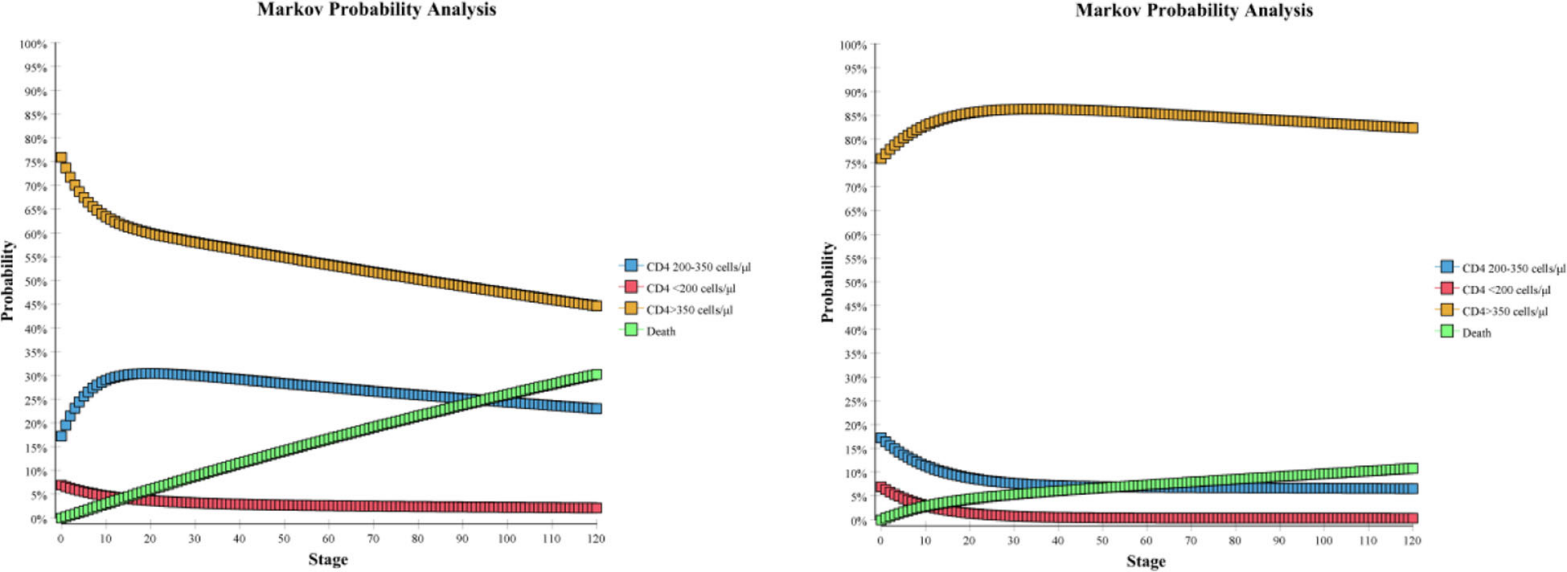
Markov cohort analysis (Left: Option B, Right: Option B+)

As shown in Table 7, the total discounted cost of ART for Option B (ART based on CD4 count eligibility) was US$ 3.32 million and for Option B+ (lifelong ART regardless of CD4 count) was US$ 23.49 million. The cost per life-year saved and QALY acquired was US$ 324.13 and US$ 348.23, respectively, for Option B and US$ 1660.69 and US$ 1764.76, respectively, for Option B+. As also shown in Fig. 4 about the results of Markov cohort analysis, Option B resulted in a 10-year survival rate of 69.7%, 10,254 life-years saved, and 9544 QALY acquired, while Option B+ resulted in a 10-year survival rate of 89.2%, 14,144 life-years saved, and 13,310 QALYs acquired.

**Table 7:** Cost-effectiveness analyses of maternal and serodiscordant partner outcomes in the long-term

Parameters	Option B	Option B+
Cost		
Program Cost (Total 10 years)	US$3,323,647.78	US$23,488,464.80
Maternal outcomes		
Number of HIV infected women alive after ten years	1394	1782
Number of life-year gained for HIV infected women in ten years	10,254	14,144
Number of QALY acquired for HIV infected women in ten years	9544	13,310
Serodiscordant transmission outcomes		
Serodiscordant partner infections averted	410	3068
Cost-effectiveness ratios		
Cost per life-year gained	US$324.13	US$1660.69
Cost per QALY acquired	US$348.23	US$1764.76
Cost per serodiscordant partner infection averted	US$8098.99	US$7655.70
ICER per life-year gained(compared to Option B)		US$5183.96
ICER per QALY gained(compared to Option B)		US$5355.42

### Sensitivity analysis for maternal and serodiscordant health outcomes

Table 8 shows the partial results of sensitivity analysis on input parameters affecting maternal and serodiscordant transmission outcomes. For maternal outcomes, Option B remained the most cost effective option with changes in discount rate, coverage rate of ART and time horizon. For serodiscordant transmission outcomes, Option B+ remained the most cost effective option with changes in discount rate, coverage rate of ART and time horizon. One-way sensitivity analysis shows the robust of model to the changes of parameters.

**Table 8:** Sensitivity analysis for maternal and serodiscordant transmission outcomes

Model parameters	Option B	Option B+
US$/Life-year averted		
Discount rate(3%)		
Best case-5%	297.93	1519.97
Worst case-0%	371.12	1914.13
Coverage rate of ART(70%)		
Best case-90%	310.05	1665.76
Worst case-45%	344.40	1652.29
Time horizon(10 years)		
Best case-20 years	305.34	1511.80
Worst case-5 years	313.27	1657.22
US$/QALY aquired		
Discount rate(3%)		
Best case-5%	320.08	1615.22
Worst case-0%	398.72	2034.09
Coverage rate of ART(70%)		
Best case-90%	332.46	1767.33
Worst case-45%	371.07	1760.39
Time horizon(10 years)		
Best case-20 years	328.15	1605.66
Worst case-5 years	336.37	1764.76
US$/Serodiscordant partner infections averted		
Discount rate(3%)		
Best case-5%	7444.31	7006.97
Worst case-0%	9273.15	8824.06
Coverage rate of ART(70%)		
Best case-90%	6567.55	5991.51
Worst case-45%	11,939.45	11,774.37
Time horizon(10 years)		
Best case-20 years	7223.82	6711.37
Worst case-5 years	8693.81	8212.97

**Fig. 5:**
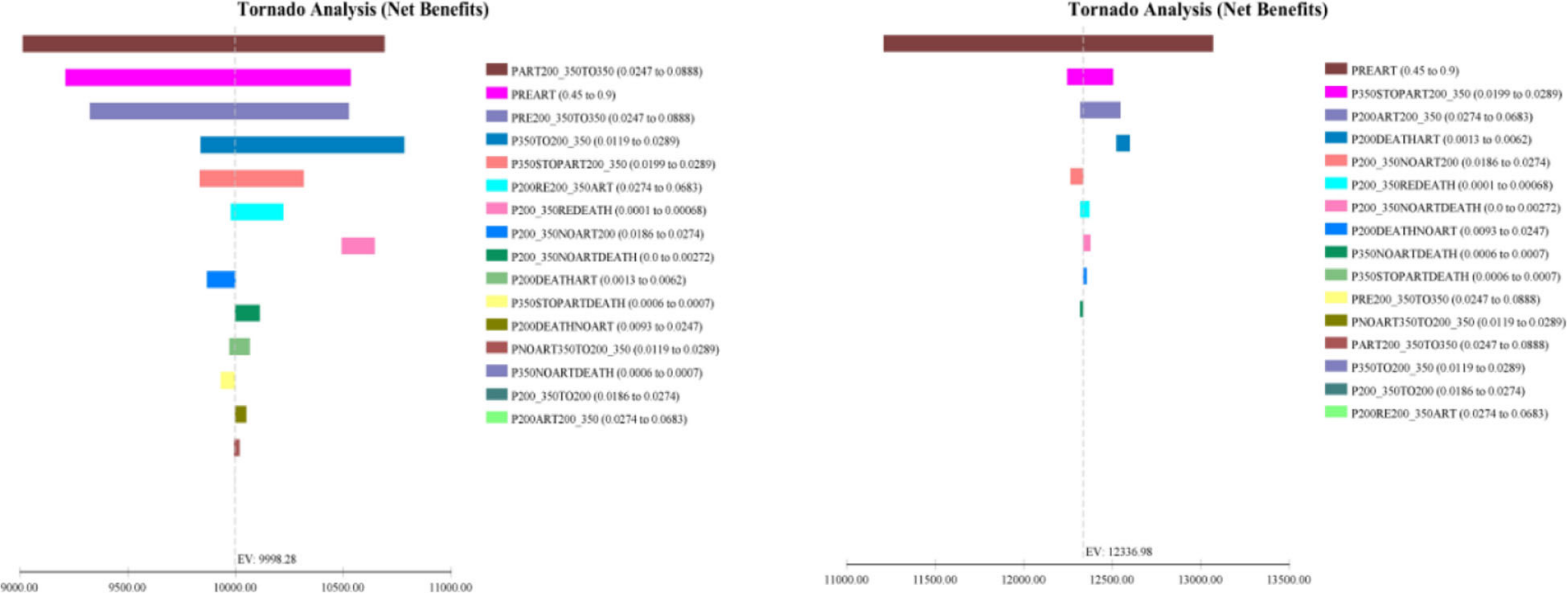
Tornado diagrams for the cost per life-year gained of Option B and Option B+

**Fig. 6:**
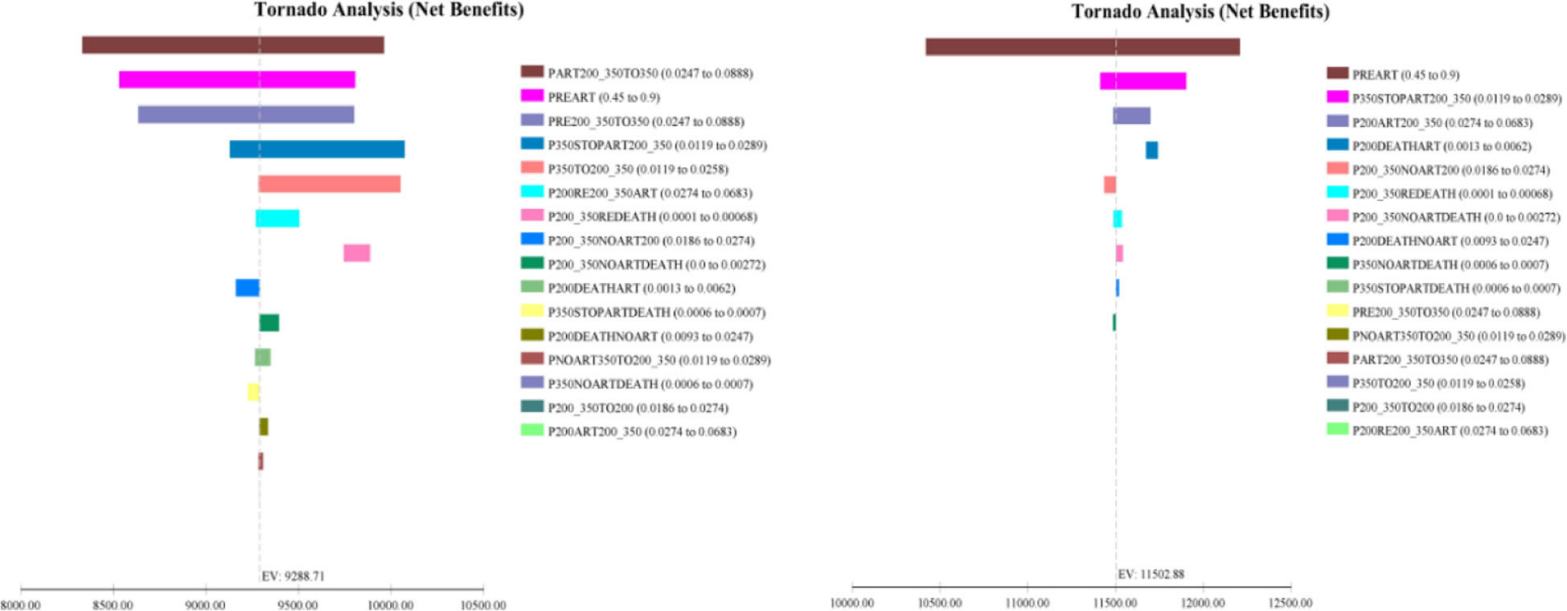
Tornado diagrams for the cost per QALY acquired of Option B and Option B+

**Fig. 7:**
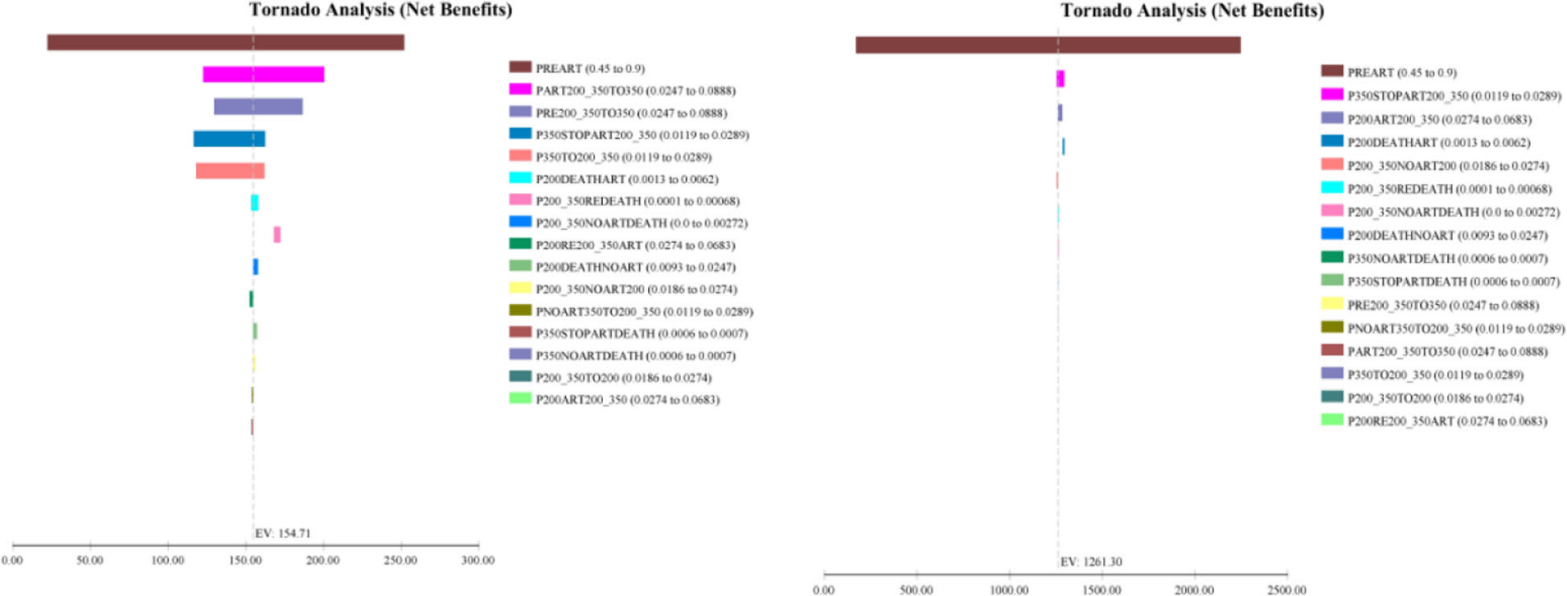
Tornado diagrams for the cost per serodiscordant partner infection averted of Option B and Option B+

As showed in Figs. 5, 6, 7, the model was most sensitive to the coverage rate of ART for Option B+. With the coverage rate of ART increasing to 90%, the ICER per life-year gained and ICER per QALY acquired compared to Option B were US$ 5149.40 and US$ 5319.36 respectively. Otherwise for Option B+, In 20-years horizon, the ICER per life-year gained and QALY acquired compared to Option B were US$3568.22 and US$3715.56 respectively.

## Discussion

In the short-term(PMTCT period), our study found that when the ART regimen transferred from Option B to Option B+,comparing with Option B, Option B+ had an less cost when getting the same effect,which demonstrated Option B+ is dominant. We consider that Option B+ responses a dominant strategy in preventing new infections among infants.

In the long-term (10 years starting from the date of PMTCT services concluded), our study found that although the total discounted cost, cost per life-year saved and cost per QALY gained for mothers of Option B+ (lifelong ART regardless of CD4)were more than Option B (ART eligibility based on CD4 count) in 10 years, Option B+ resulted in a survival rate of 89.2%, a better survival outcomes than Option B, with a survival rate of 69.7%. For their partners at 10 years, Option B+ resulted in a cost of US$ 7655.70 per infection averted, which was lower than Option B. If we considered the benefits of the maternal survival and serodiscordant infections, Option B+ could show a higher cost-effective. Otherwise the ICER per life-year gained and QALY acquired was US$5183.96 and US$5355.42 respectively, when compared to Option B for mothers. Using the WHO standards for determining the cost-effectiveness of a strategy [3], the ICER per life-year saved and QALY acquired of Option B+ was less than the 3 × Yunnan’s per capita GDP(US$13,000) threshold, we found that Option B+ responses a cost-effective strategy in keeping mother alive and preventing new serodiscordant infections among their partners.

The sensitivity analyses verified the robustness of our cost-effective measures after changing a variety of parameters. In the short-term, by the increasing of efficacy of Option B/Option B+ in reducing transmission rates, the cost per infant infection averted and the cost per QALY acquired of Option B + decreased slightly, especially as the goal of elimination of mother-to-child transmission of HIV achieved, Option B+ would be more attractive.In view of the lifetime ART(69.5 years) for the infected children, when the lifetime ATR cost was include into the total cost, Option B+ could dominate the most-effective option to prevent new infant infections in Yunnan Province. In the long-term, sensitivity analyses showed the cost-effective of life-year gained, QALY acquired and serodiscordant infection averted were most sensitive to the coverage of ART. Expanding the coverage of ART to those eligible women appears to be more cost-effective, particularly when the coverage rate of ART researches to 90%, the ICER per life-year gained and per QALY acquired compared with Option B decreased, with US$ 5149.40 and US$ 5319.36 respectively. Especially with the year increasing to 20 years, to implement Option B+ will demonstrate the priorities, all the ICER per life year gained and per QALY acquired have declined less than 1 × GDP, with US$ 3568.22 and US$ 3715.56 respectively. From a comprehensive and long-term perspective, Option B+ would be the optimal strategy in Yunnan province when the goals of “90–90-90” (90% of all people living with HIV will know their HIV status, 90% of all people with diagnosed HIV will receive sustained antiretroviral therapy and 90% of all people receiving antiretroviral therapy will have viral suppression.)and “95–95-95” (95% of all people living with HIV will know their HIV status, 95% of all people with diagnosed HIV will receive sustained antiretroviral therapy and 95% of all people receiving antiretroviral therapy will have viral suppression.) achieved.

In comparison with previous cost-effectiveness studies in low- and middle- income countries, less differences were observed. All the previous studies demonstrated the cost effective of Option B+. A previous modeling study of mother-to-child transmission of HIV in Ghana [26], suggested that Option B+ is a cost-effective use of limited resource. In Uganda, lifelong ART was associated with highly cost-effective for prevention mother-to-child transmission of HIV and acquiring additional public health benefit [27]. In Malawi, when averted infants infections and maternal survival outcomes were considered together, Option B+ represented a more cost-effective policy option [2]. In Nigeria, lifelong ART could provide the greatest incremental benefit to prevent HIV transmission among HIV serodiscordant couples [25]. Our study comprehensively included the benefits of infants, mothers and serodiscordant partners together, overcoming the deficiency of the previous cost-effective analyses of Option B+ to omit the important benefits [11]. In China, there is few study to model the cost-effectiveness evaluation of Option B+. Option B+ is recently endorsed by WHO to scale up the PMTCT programs in countries with high-burden [10]. Our study is the first study to develop the cost-effectiveness models informed by the field data of Yunnnan, China and addressed the potential cost saving associated with Option B+ from short-term and long-term. We hope our study could assist the province to complete a policy switching from previous strategies to Option B+ and supply scientific evidences for the applicability of Option B+ in Yunnan province. Future research efforts in the cost-effective analyses of Option B+ should be directed at providing more evidences in different areas in China and also across different cultures. We believe our study could supply a useful framework to other similar studies.

Our study had some important limitations. Firstly, most the parameters included in the models are based on the Yunnan context. For example, Yunnan province is with the highest HIV prevalence level in China, different HIV prevalence level could address different cost-effective results of Option B+. So applying the results elsewhere should be with more cautions. Secondly, our study adopted a perspective of health care system, because the direct cost is the major component of total cost of implementation of Option B+. A further study should be conducted by using the perspective of societal prevention so that Option B+ could be more preferable as more evidences indicated the possible influence to the family and society. Thirdly, our study didn’t consider the other factors associated with decision making regarding the resource allocation. These factors may include society and culture factors.

## Conclusion

In conclusion, we have presented an economic analysis that provides evidence that HIV PMTCT Option B+ is a cost-effective strategy for comprehensive HIV prevention for infants and serodiscordant partners and life-long treatment for mothers in Yunnan Province, China. Option B+ could be implemented in Yunnan province, especially as the goals of eMTCT(MTCT< 2%) and “90–90-90”(coverage rate of ART> 90%) achieved, Option B+ would be more attractive.

## Additional file

Additional file 1: Appendix The process of decision model construction the model input parameters and process of decision tree and Markov decision tree model. (DOC 29 kb)

## Abbreviations

ANC: Antenatal care
ART: Anti-retroviral therapy
GDP: Gross domestic product
ICER: Incremental cost-effectiveness ratio
PMTCT: Prevention of mother-to-child transmission
QALY: Quality-adjusted life-years
WHO: World Health Organization
