# Supplementary material for: Cost-effectiveness of option B+ in prevention of mother-to-child transmission of HIV in Yunnan Province, China
Source: BMC Infect Dis. 2019 Jun 11;19:517. doi: 10.1186/s12879-019-3976-5 (PMC6560771; doi:10.1186/s12879-019-3976-5)
Supplement: Supplementary file 1 — Appendix The process of decision model construction the model input parameters and process of decision tree and Markov decision tree model. (DOC 29 kb) [file 12879_2019_3976_MOESM1_ESM.doc]

**The process of decision model construction**

- **The process of simple decision tree construction**

**Model input parameters**

**Maternal characteristics**

Based on the data from Yunnan maternal and infant health care hospital, a hypothesis cohort of HIV-infected women was estimated with the number of 2,000 (720,435 pregnant women were tested and HIV prevalence rate in pregnant women was about 0.3%). The mean age of pregnant women with HIV at first ANC visit was 25 years. Mean CD4 count was 445 (36% of women with CD4 count≤350 cell**/**μl ). Among them 1120 chose to delivery (the termination rate was about 44%).

**MTCT risks and Infant Life expectancy Estimated**

Because the formula-feeding rate in Yunnan Province was nearly 100%, we mainly considered the risk of MTCT during pregnancy. We used the risk of MTCT during pregnancy reported by Yunnan Maternal and Infant Health Care Hospital from 2003 to 2015. We adopt 70.5 reported by Yunnan Provincial health and family planning commissionin 2015^[1]^ as the life-expectancy estimates of infant without HIV infection and adopted 69.5 reported by previous study as the life-expectancy estimates of people living with HIV infection under the background of cART ^[2]^.

**Cost Inputs**

The data of cost estimates were obtained directly from Yunnan Maternal and Infant Health Care Hospital. All costs were shown in US dollars in 2016. The cost of rapid HIV testing was US$4.06 per test and all pregnant women were assumed to undergo at least one HIV test during the first antenatal visit for Option A, Option B and Option B+ . Option A and Option B to determine the eligibility for ART at a cost of US$ 13.06 per test and 4 times in the PMTCT period. Costs of ARVs for Option A included 3 months during pregnancy and one week after delivery and for Option B included 6 months during pregnancy and 42 days after delivery. The costs of drugs per women receiving Option A for 3 months of prophylaxis was US$ 42.08 (consisting of US$23.94 for maternal ARVs prophylaxis, US$18.14 for infant ARVs prophylaxis). The cost of drugs per woman receiving B and B+ in the PMTCT programs was US$253.79 per woman (consisting of US$235.65 for maternal ART, US$18.14 for infant ARVs prophylaxis).

Other costs for all the options included formula feeding, infant diagnosis testing and safety delivery. Nearly 100% of all infants born to an HIV-infected mother were assumed to be tested with DNA PCR at a cost of US$ 26.12 and be formula feeding at a cost of US$580.42. The average cost of delivery was US$290.21(the rates of vaginal and cesarean was 50% and the cost of vaginal and cesarean was US$145.10 and US$435.31 respectively). The stillbirth equaled to 0.45 pediatric infection.

- **The process of Markov decision tree construction**

**Model parameters**

**Transmission model parameters**

The primary probabilities were estimated from distribution rate before the starting of Markov process. We used the CD4 count distribution of the last testing in the PMTCT period as the primary probability. Death rates, sexual transmission rates and CD4 count transmission probabilities by CD4 counts and ART status were estimated from data from other published literature. All rates were converted to monthly probabilities. Data on sexual behavior and serodiscordant partner characteristics came from the studies conducted elsewhere in China. Efficacy of TasP and ART based on CD4 counts were taken from other studies.

**Cost parameters**

Full costs (in US$ 2016) of programs implementation were estimated from provider perspective and life-time ART cost computed from a costing study conducted in China^[3]^. The costs mainly comprised of the cost of ART, the cost of regular testing and following up, the cost of reaction treatment and the cost of opportunistic infection prevention.

**Intervention scenarios**

The main baseline scenario was offering ART to the HIV infection women at CD4≤350 cells/μl when ended the PMTCT services. The intervention scenarios considered were TasP(offering ART to all the HIV infection women lifelong whatever the CD4 counts). We assumed the current ART coverage level (70%) ^[4]^of Yunnan Province among eligible HIV infection women.

**Model analysis**

The impact of each scenario was estimated in comparison with each other in terms of maternal life expectancy from delivery, serodiscordant partner infections averted and Quality Adjusted life-years (QALYs) acquired over 10 years. QALYs were calculated by summing up person-years spent in different CD4 counts and ART categories and the QALY weights in the different health status were estimated by Global Burden of Disease Study in 2010 ^[5]^. Cost-effectiveness was calculated as the incremental cost per life-year gained and QALY acquired and infection averted over 10 years. The costs were discounted into the future at a rate of 3% per year. The cost-effectiveness threshold used were 1×GDP and 3×GDP per life-year and QALY acquired for highly cost-effective and cost-effective intervention, respectively.

**References**

[1] Yunnan.cn. Yunnan Provincial health and family planning commission: The mean life ecpectancy of Yunnan province was to 70.5 years old in 2015. <http://yn.yunnan.cn/html/2016-02/26/content_4195182.htm>. 2016-02-06/2016-11-28.

[2] II. Demographic impact of AIDS. <http://www.un.org/esa/population/publications/AIDSimpact/5_CHAP_II.pdf.> 2017-11-02.

[3] Zhiwei G. The direct medical costs analysis of natioanl free antiretroviral treatment for HIV/AIDS patients. Beijing: Chinese academy of medical sciences&Peking union medical college, 2008.

[4] Yunnan net.the patients with HIV alive more than 90,000 in Yunnan Province

http://yn.yunnan.cn/html/2016-11/30/content_4636269.htm.2016-11-30/2017-01-18.

[5] Salomon JA, Vos T, Hogan DR, et al. Common values in assessing health outcomes from disease and injury: disability weights measurement study for the Global Burden of Disease Study 2010. Lancet 2012;380:2129-2143.
